# X-ray vision in 2025

**DOI:** 10.1016/j.igie.2025.07.001

**Published:** 2025-07-05

**Authors:** Linda S. Lee, Brian Fleming

**Affiliations:** 1 Brigham and Women's Hospital, Boston, Massachusetts, USA; 2 Omega Medical Imaging, Sanford, Florida, USA

## Editor's introduction

Enrico Salvioni and Thomas Edison invented the fluoroscope in 1896.^1^ Unfortunately, Thomas Edison's assistant, Clarence Dally, died in 1904 of cancer as a result of radiation exposure from the fluoroscope. Strides have been made since then in protecting both patients and staff from radiation exposure. Fluoroscopy has been an integral part of gastroenterology beginning with barium contrast studies in the early 1900s and remains an essential component of advanced endoscopy procedures including endoscopic retrograde cholangiopancreatography and therapeutic endoscopic ultrasound. However, fluoroscopy systems have been designed for radiology and only 1 company, Omega Medical Imaging, has designed a system with the interventional endoscopist in mind.

I am delighted to share my discussion with Brian Fleming, who is President and CEO of Omega Medical Imaging. Brian has more than 30 years of leadership experience in the medical imaging and biotechnology sectors. For the past 15 years, he has led Omega Medical Imaging as President and CEO, driving innovation and growth in a rapidly evolving industry. Brian holds an undergraduate degree from the Rochester Institute of Technology and an MBA from Syracuse University. A proud father and grandfather, he is passionate about radiation safety and advancing health care technologies while nurturing the next generation of industry leaders.

### Linda Lee (LL)

**Figure 1:**
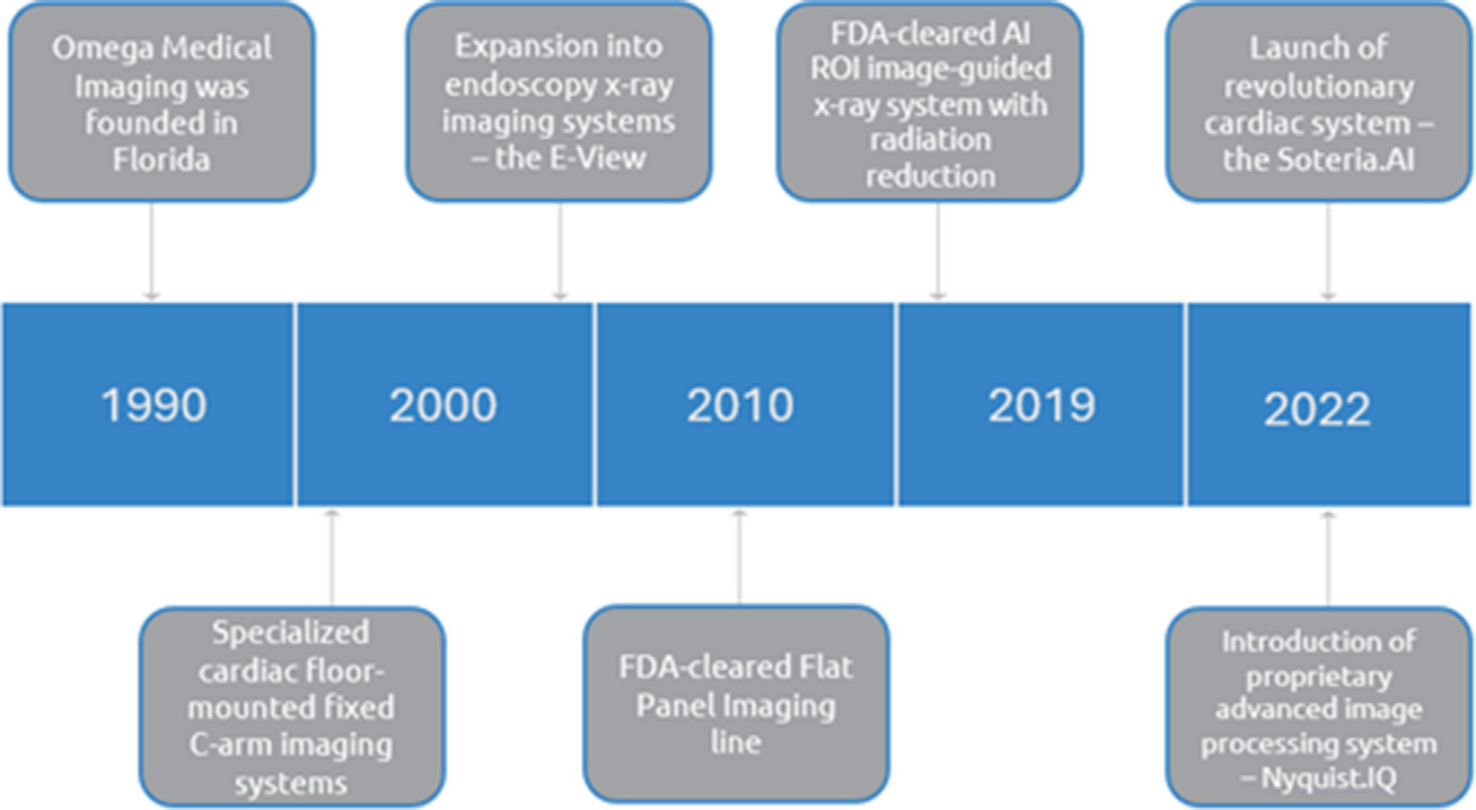
Timeline of key events at Omega. *AI ROI*, Artificial intelligence region of interest; *FDA*, U.S. Food and Drug Administration.

Would you share with us the history of Omega (Fig. 1)?

### Brian Fleming (BF)

Founded in 1990, Omega Medical Imaging initially focused on catheter laboratories and found success in providing x-ray systems dedicated to the electrophysiology and interventional cardiology fields. In 2000, Omega developed a more specialized cardiac Floor-Mounted Fixed C-Arm imaging system for performing catheterization and basic angiographic procedures in hospitals and private practice offices. That same year, Omega embarked on another expansion of its imaging systems to include endoscopy x-ray imaging. In 2010, the U.S. Food and Drug Administration (FDA) cleared Omega's new Flat Panel Imaging line. In 2019, Omega became the first, and remains the only, FDA-cleared manufacturer to integrate an artificial intelligence (AI) region of interest (ROI) radiation-reduction technology in an image-guided x-ray system. This AI ROI system, FluoroShield, was demonstrated to significantly reduce radiation exposure in the endoscopy laboratory without impacting image quality. In 2022, Omega launched a revolutionary cardiac system—the Soteria.AI— and the industry's most advanced proprietary image processing system called Nyquist.IQ, along with FluoroShield.

**LL**: How did Omega develop the only fluoroscopy system for gastroenterology? Would you review the key steps and challenges along the way?

**BF**: The journey to developing the world's only FDA-cleared, fixed fluoroscopy system specifically designed for interventional endoscopy (IE) procedures was driven by a combination of clinical collaboration, technical innovation, and a commitment to advancing safety standards in x-ray medical imaging.

## Origins and clinical collaboration

Our story began in 2000, when a visionary interventional endoscopist approached us with a challenge: Could we adapt a catheter laboratory–style, floor-mounted C-arm x-ray system to better serve the unique demands of IE? At that time, IE was a relatively new specialty, and most endoscopy units relied on mobile C-arms or radiographic/fluoroscopic tables and technologies not optimized for the complex workflows and imaging needs of advanced endoscopic procedures such as endoscopic retrograde cholangiopancreatography.

We worked closely with leading IE physicians to understand their clinical requirements, including the need for real-time, high-quality imaging, ergonomic access, and enhanced radiation safety. This partnership was essential in designing a new system from the ground up that truly supported the workflow and safety of IE teams.

## Key development steps

• Needs assessment and customization: By engaging directly with clinicians, we identified gaps in existing imaging solutions and tailored our system—eventually known as the E-View—to address them. This included optimizing the imaging, table and shielding, C-arm design, and user interface for IE procedures.
• Engineering and regulatory hurdles: As the only manufacturer building a fixed, floor-mounted C-arm for IE, we faced a lack of industry standards and regulatory pathways specific to this application. Navigating FDA clearance required rigorous risk analysis and validation, and a focus on patient and staff safety.
• Education and market adoption: Many hospitals and administrators were unfamiliar with the unique imaging needs of IE and were hesitant to invest in new contract-exempt solutions. We partnered with physician champions to educate stakeholders on the clinical benefits and long-term value of dedicated IE fluoroscopy systems.

## Challenges and looking forward

• Awareness and standardization: One of our ongoing challenges is raising awareness about radiation risks and the benefits of AI ROI technology among all stakeholders in the IE laboratory. Omega is committed not just to selling more systems, but to setting a new standard for radiation safety in fluoroscopy.
• Continuous improvement: Omega continues to collaborate with clinicians, engineers, and industry partners to refine our technology and advocate for its widespread adoption as a new baseline for interventional x-ray imaging.

Omega's journey has been defined by listening to clinicians, solving real-world problems, and pushing the boundaries of what is possible in gastrointestinal imaging. By bringing together clinical insight, technical expertise, and a relentless focus on safety, we developed a system that not only advances patient care but also sets a new benchmark for x-ray imaging.

**Figure 2:**
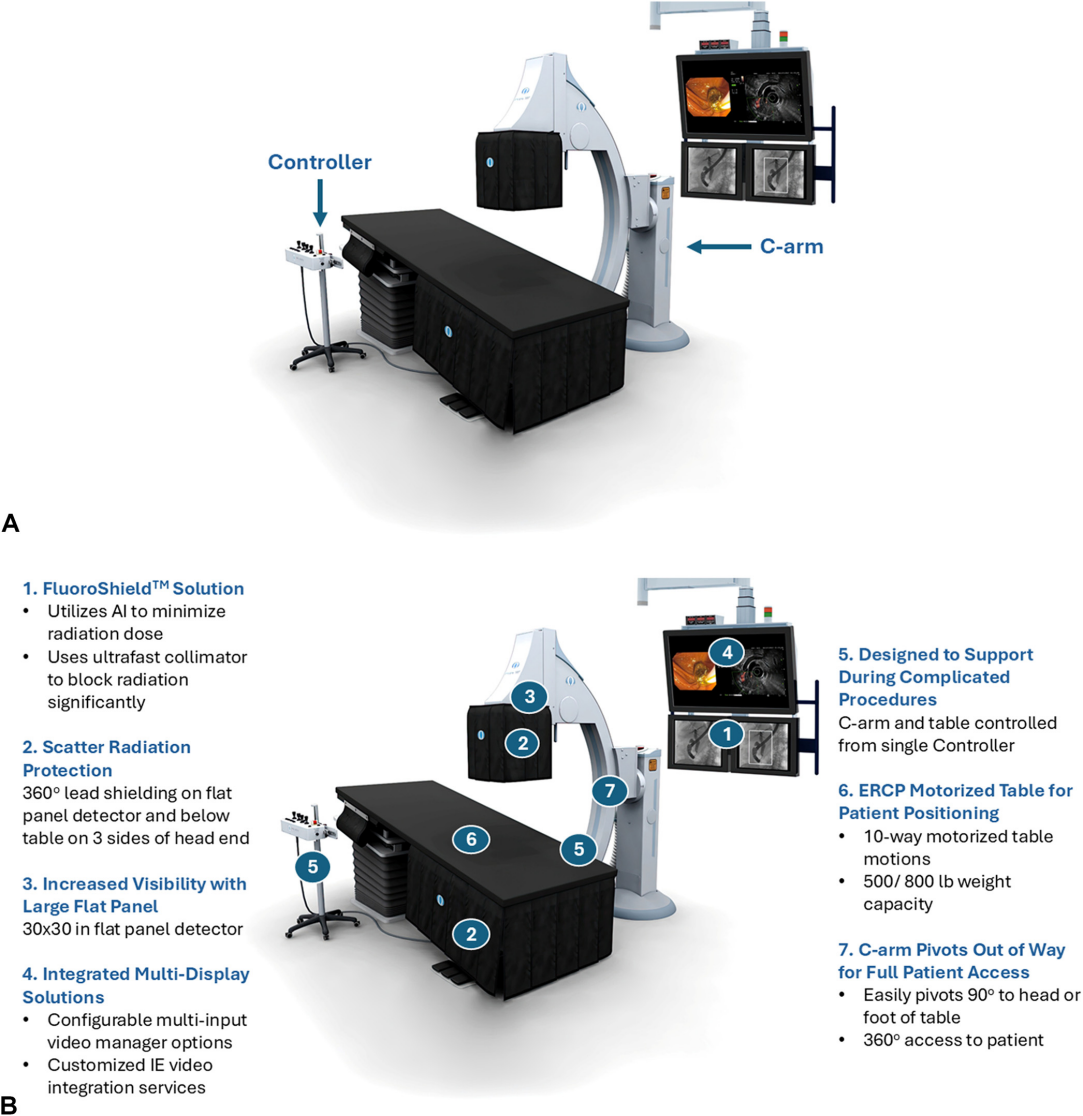
**A,** Omega system with monitors on the right, fixed C-arm (*C-arm arrow*) and table, and control for fluoroscopy (*Controller arrow*). **B,** Key features of Omega system. *AI*, Artificial intelligence; *ALARA*, as low as reasonably achievable, *HD*, high definition; *IE*, interventional endoscopy.

**LL**: What makes the Omega system unique compared with other fluoroscopy systems (Fig. 2)?

**BF**: Unlike conventional fluoroscopy systems originally designed for orthopedics or general radiography, the Omega Fixed Floor Mounted C-arm is purpose-built for complex IE procedures. Every aspect, from its ergonomic design to its imaging workflow, has been optimized to meet the specific demands of interventional endoscopists—supporting challenging cases with intuitive controls and superior image quality.

The Omega system features AI-powered ROI radiation reduction technology (FluoroShield). This system is unique because it is the only FDA-cleared fluoroscopy platform specifically engineered for IE, featuring AI-driven ROI technology that dramatically lowers radiation exposure without compromising image quality or workflow.

Despite the dramatic reduction in radiation, the Omega system delivers outstanding image quality. Proprietary algorithms ensure high-resolution, detailed images so fine guide wires, stones, etc, can be seen, even in larger patients—better supporting accurate diagnosis and therapy even at reduced-dose settings.

Built-in measurement tools allow the physician to measure strictures, stones, and anatomy to better determine the next course of action during cases. The C-arm and table are designed for ease-of-use—allowing independent movements and efficient patient positioning and drainage. In addition, the C-arm easily swings out of the way when x-ray is not needed or in the case of an emergency.

Large, high-definition medical displays and configurable video integration support the visualization needs of advanced endoscopic procedures. With a large 30- × 30-cm flat panel, the system provides a wide field of view, essential for complex therapies and anatomical visualization.

**Figure 3:**
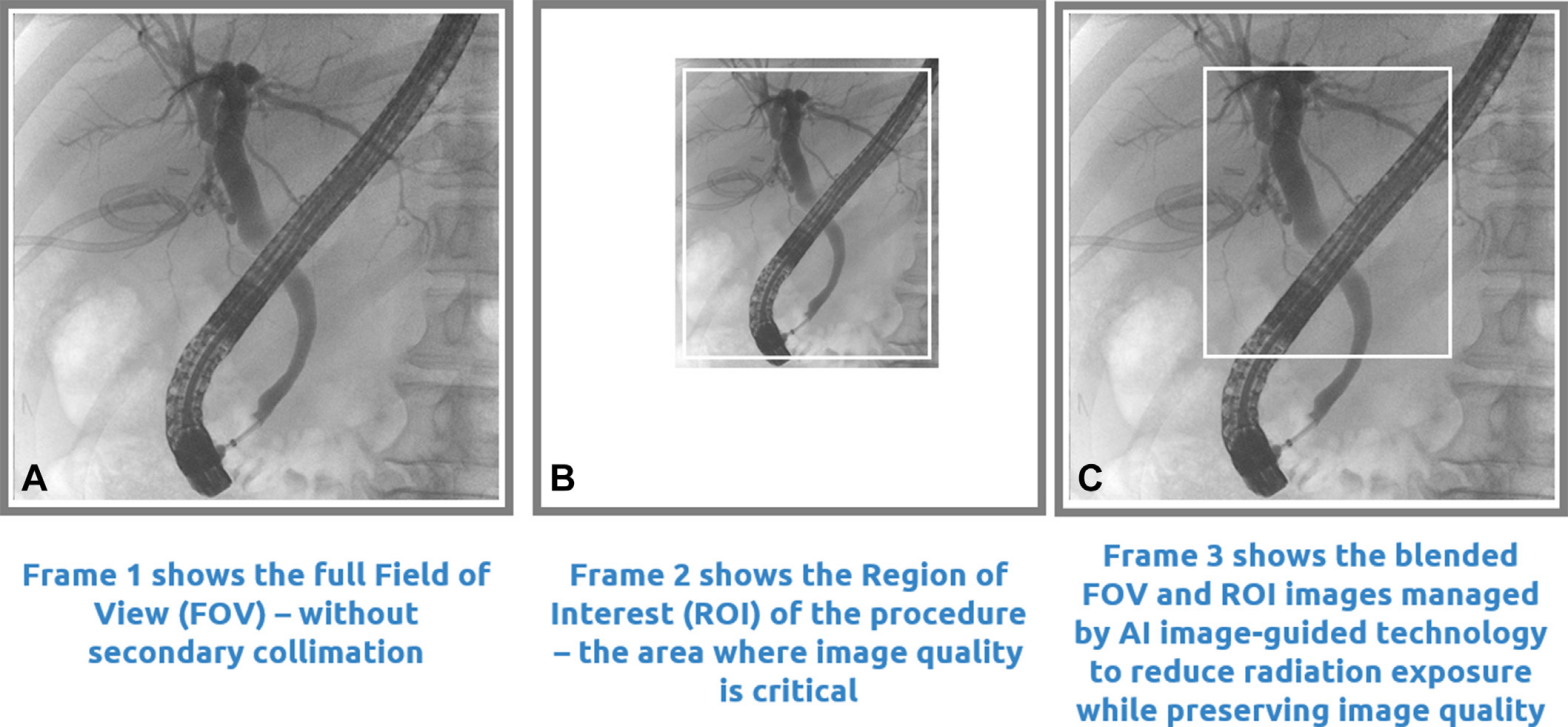
**A,** Full field of view (FOV) without collimation; (**B**) collimated region of interest (ROI) only; (**C**) artificial intelligence (AI) blended image with ROI collimated while maintaining full FOV and significantly lower radiation dosage to the area outside ROI.

**LL**: Would you discuss the AI system to reduce radiation exposure and how it works (Fig. 3)?

**BF**: A major challenge in fluoroscopy-guided gastrointestinal procedures is radiation exposure to doctors, patients, and staff. Recognizing this, Omega invested in AI-powered ROI technology, inspired by peer-reviewed research demonstrating its ability to significantly reduce radiation dose without compromising image quality or workflow.

Unlike conventional systems that irradiate the entire field of view, AI ROI technology continuously identifies and tracks the anatomical ROI, dynamically adjusting collimation and dose-area product exposure. This ensures that only the area requiring imaging receives the full x-ray dose, while the surrounding tissues and everyone in the procedure room are better protected.

## How AI ROI technology works

### Automatic ROI detection

During a procedure, the AI system continuously analyzes the live x-ray imaging to identify where the interventionalist is working in real time. This is achieved through advanced image analysis algorithms that track instruments and anatomical landmarks in real time.

**Impact**: Peer-reviewed studies and real-world use have shown that AI-guided ROI collimation can reduce patient radiation exposure by more than 60%, with a comparable reduction in scatter radiation to staff—far exceeding the reductions achieved by conventional dose-saving features alone. This represents a leap forward in safety, akin to how pulsed fluoroscopy, frame-rate reduction, and collimation became standards in the industry. A prospective study in advanced endoscopy showed a 59.4% reduction in dose-area product to patients and a 61% reduction in scatter to staff when using AI-enabled fluoroscopy compared to conventional systems.^2^ Importantly, these reductions were achieved without sacrificing image quality.

### Dynamic collimation

Once the ROI is identified, an ultra-fast secondary collimator automatically shapes and restricts the x-ray beam to this area, updating up to 30 times per second. The peripheral anatomy outside the ROI receives a much lower radiation exposure at a lower frame rate, as the system intermittently opens the collimator to capture a full-field image for orientation. We continually improve the ROI tracking and optimize the dynamic collimation by training the AI on clinical images.

### Image blending

The system seamlessly integrates the high-resolution, real-time image of the ROI with a lower frame rate, full field-of-view “road map.” This blended display ensures clinicians have the anatomical context they need without compromising image quality and reducing radiation exposure to everyone in the room.

### Consistent, hands-free protection

The ROI technology operates automatically, integrating seamlessly into the clinical workflow without requiring manual adjustments or interrupting the procedure. The result is consistent, repeatable radiation reduction, regardless of operator technique or experience. Manual setting of the ROI is also available.

Reduced scatter radiation benefits everyone, including the doctors and staff in the procedure rooms, addressing a critical occupational health concern. Lower radiation dose also reduces the risk of both immediate and long-term adverse events for patients.

## Setting a new standard in radiation safety

• The Omega system's AI-guided ROI technology is not just an incremental improvement—it is a paradigm shift in radiation protection. By making advanced radiation reduction automatic and practical for real-world clinical use, Omega sets a new benchmark for safety in interventional imaging. This approach aligns with the ALARA (as low as reasonably achievable) principle and represents a model that, ideally, will become standard across all x-ray systems in the future.
• Our vision is that ROI radiation reduction—much like pulsed fluoroscopy, frame-rate reduction, and collimation—will soon be standard on all interventional x-ray systems.

**LL**: How long was the process for FDA approval and what were the biggest challenges with this?

**BF**: The concept of using ROI techniques to enhance radiation protection in interventional x-ray imaging has been discussed in academic literature for more than 25 years. However, the technology required to realize this vision—particularly the integration of AI, rapid electromechanical collimation, and advanced image processing—has only recently become viable.

Bringing this innovation from concept to clinical reality required a multidisciplinary approach. Our team combined engineering expertise, precise craftsmanship, and cutting-edge AI algorithms to ensure that the system not only delivered substantial radiation reduction but also maintained the uncompromised image quality demanded by clinicians.

Navigating the FDA clearance process for a Class II medical device is always a rigorous undertaking, with timelines typically ranging from 3 to 6 months for well-prepared 510(k) submissions. Our experience was that the FDA recognized the real-world benefits and robust safety profile of AI-driven ROI technology. We achieved 510(k) clearance in just over 4 months, a testament both to our preparation and to the FDA's commitment to advancing innovations that improve patient and staff safety.

The greatest challenge was not technical or regulatory, but cultural: introducing a paradigm shift in how radiation safety is approached in interventional imaging. Our hope is that—much like pulsed fluoroscopy and collimation before—ROI-based radiation reduction will soon become a standard feature across all x-ray systems.

**LL**: What improvements have Omega made to the system?

**BF**: Omega is continually evaluating and improving our technology and the systems we build. Much of that evaluation is born from the feedback and insights of the physicians who use our systems every day. As the procedures they perform evolve, so too must the systems they rely on. Input from physicians and staff led to improvements in our systems (for example, adding lead shielding above the table to reduce scatter radiation; the ability to pivot the C-arm out of the way for ease-of-access to the patient and for table use during non-fluoroscopy cases; and design of table [added width and increased weight capacity up to 800 pounds]).

**LL**: What has been the strategy for Omega marketing its system in the United States and globally?

**BF**: Omega's strongest marketing strategy has always been more than a strategy; it's been about building awareness, trust, and relationships with the doctors and hospitals we serve. We work closely with the physicians who need and will use our systems. Often, these physicians are referred to Omega by other physicians who have implemented the use of our AI ROI E-View systems into their practice. We will build on these relationships and learn new ways we can innovate to better support those doctors and their patients.

Omega has entered non-U.S. markets in Europe, Asia, and the Middle East. Our approach to these markets did not change, but there were added challenges with regulatory pressures as well as import fees and duties.

**LL**: What does the future hold for Omega?

**BF**: Hopefully, we will continue to earn the trust of current and future customers. On the basis of their suggestions, we continue to innovate new solutions for the challenges they and their staff face in delivering care to their patients. We will continue to educate about radiation safety and hope ROI technology will soon become a standard feature across all x-ray systems—benefiting doctors, staff, and patients worldwide. Ultimately, widespread adoption of this technology will drive a new era of safety and efficacy in interventional procedures.

## Editor's closing remarks

Despite EUS overtaking much of therapeutic endoscopy, fluoroscopy remains an integral part of gastroenterology. Achieving highest image quality combined with lowest radiation exposure to patient and providers is most important. Omega Medical Imaging has endeavored to meet these goals for the interventional gastroenterologist by leveraging AI technology. Radiation safety continues to be a salient issue, and proper training and education of fellows and endoscopists are essential to ensuring that they understand how to achieve the best image needed for procedures while minimizing radiation exposure to everyone in the procedure room.^3^

## Disclosure

The following authors disclosed financial relationships: B. Fleming: Full-time employee/Owner: Omega Medical Imaging LLC. L.S. Lee: Consultant: Fujifilm Healthcare Americas, Microtech, Cook Medical, and Boston Scientific; research support: Medtronic.
